# A Cu(II)-based coordination polymer: catalytic properties and treatment activity on stroke

**DOI:** 10.1080/15685551.2022.2086396

**Published:** 2022-06-09

**Authors:** Peng-Ju Gao, Ji-Rong Liu

**Affiliations:** a Neurology Department, Xingyuan Hospital of Yulin, Yulin, Shaanxi, China; b Neurology Department, Weinan Central Hospital, Weinan, Shaanxi, China

**Keywords:** Cu(II) compound, infinite chain, Mixed-ligand, catalysis, stroke

## Abstract

A fresh Cu(II) coordination polymer, i.e., [Cu_3_(Hmbdc)_2_(mbdc)_2_(dmphen)_2_]_n_ (**1**, H_2_mbdc = isophthalic acid, dmphen = 4,7-dimethyl-1,10-phenanthroline), has been generated with the hydrothermal reactions between Cu salts and the mixed ligands of 4,7-dimethyl-1,10-phenanthroline and isophthalic acid. Moreover, the catalytic activity of **1** was evaluated *via* degrading the Congo red with a method of Fenton with an excellent degradation efficiency of 95.8% at 100 min. Next, the application value of compound on stroke was assessed, and the related mechanisms were explored at the same time. First of all, the tumor necrosis factor-α and recombinant rat IL-1β content released into the plasma were determined with enzyme-linked immunosorbent assay detection kit. Besides, the activation of the HMGB1/TLR4 signaling pathway activation in cerebral vascular endothelial cells was also determined with real-time reverse transcription–polymerase chain reaction assay.

## Introduction

Stroke is a major disease that threatens human health and safety of life and property. Stroke is the loss of neurons due to cerebrovascular wall lesions or blood flow disorders. Inflammation is the defense response of the body with the vascular system to damage factors, and it is a comprehensive process of damage, anti-injury and repair [1]. In foreign studies, it was found that a large number of CRP deposits were found in the pathological anatomy of atherosclerotic plaques, and a large number of complement terminal-reactive protein C5b-9 deposits were seen in the lesions, suggesting the important role of inflammatory response during the development of stroke [2].

**Scheme 1.**
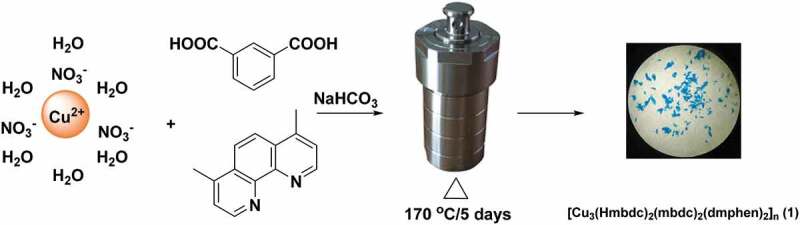
The synthetic route for complex **1.**

Coordination polymers (CPs) as a new type of hybrid materials have great application prospects in catalysis, heterogeneous luminescence, ion exchange, gas separation and storage and other areas owing to their high surface areas and tunable structures [3–6]. For the purpose of developing new functional materials, a myriad of CPs has been successfully constructed *via* various synthetic strategies [7–11]. Among a variety of the strategies, the method of dual-ligand is one of the most suitable and effective ways to establish the CPs having high thermal stabilities and promising properties [12–16]. In contrast to the single ligand, dual ligand with carboxylate ligand and N-containing ligand not only can effectively tune the structural diversities *via* the synergistic coordination effect but also can endow CP’s better performances from the combination of dual ligand [17]. Therefore, based on the aforementioned aspects, in the experiment, we employed the method of dual ligand to establish the new hybrid materials. The combination of isophthalic acid (H_2_mbdc) and 4,7-dimethyl-1,10-phenanthroline (dmphen, Scheme 1) was selected as the organic building blocks owing to diversified coordination modes of H_2_mbdc ligand and strong chelating ability of dmphen. Via the hydrothermal self-assemble reactions of Cu(II) ions, H_2_mbdc and dmphen, we acquired a novel coordination polymer in success, i.e, [Cu_3_(Hmbdc)_2_(mbdc)_2_(dmphen)_2_]_n_ (**1**, H_2_mbdc = isophthalic acid, dmphen = 4,7-dimethyl-1,10-phenanthroline). Furthermore, complex **1ʹ**s catalytic activity for the Congo red degradation in a reaction of Fenton was also investigated. In the biological section, the ELISA assay and real-time RT-PCR assay were conducted, and the application value of the compound on stroke was assessed.

## Experimental

### Materials and instrumentation

All of the raw materials employed in our investigation were obtained from the market, and they were exploited without processing. Through utilizing the analyzer of elemental Vario EL III, the hydrogen, nitrogen and carbon elements were analyzed. The PXRD could be analyzed with the powder diffractometer of PANalytical X’Pert Pro utilizing the Cu/Kα radiation (with λ of 1.54056 Å) with 0.05° step size. The thermogravimetric analysis were conducted via exploiting the thermoanalyzer of NETSCHZ STA-449C under the atmosphere of N_2_ with 10°C/min rate. For the solution of Congo red, its ultraviolet–visible absorption spectra could be harvested with the ultraviolet/visible spectrophotometer of Perkin-Elmer Lambda 900.

#### Synthesis of compound [Cu_3_(Hmbdc)_2_(mbdc)_2_(dmphen)_2_]_n_ (1)

The mixture synthesized by 0.3 mmol of Cu(NO_3_)_2_.3H_2_O, 0.06 g and 0.2 mmol of H_2_mbdc, 0.1 mmol of dmphen, 0.1 mmol of NaHCO_3_ along with 12 mL of H_2_O was kept into a stainless steel container with Parr Teflon lining (25 mL), and after that, the connector was sealed, and it was then heated to 170°C and maintained at 170°C temperature for 5 days. The complex **1ʹ**s blue massive crystals were gained after naturally cooling to environmental temperature with the yield of 48% according to dmphen. Elemental analysis calcd. (%) for the complex **1** C_60_H_42_Cu_3_N_4_O_16_: N, 4.42%; H, 3.32% and C, 56.89%. Found (%): N, 4.46, C, 56.93 and H, 3.28%. Elemental analysis calcd. (%) for the complex **1** after dye degradation: N, 4.42%; H, 3.32% and C, 56.89%. Found (%): N, 4.38, C, 56.61 and H, 3.55%. FT-IR (4000–400 cm^−1^): 3414 (brs), 3081 (w), 2930 (w), 2117 (s), 1632 (s), 1570 (s), 1447 (s), 1353 (s), 1095 (w), 1024 (w), 930 (w), 889 (w), 776 (s), 693 (w), 415 (s).

#### X-ray crystallography

The compound **1ʹ**s data of single crystal have been harvested by the graphite–monochromated Mo–*Kα* radiation (with *λ* of 0.71073 Å) via employing the diffractometer of Mercury CCD at 293(2) K. Dual direct approach was applied to solve the compound’s architecture utilizing the *ShelxT*, and then *SHELXL*-2014 was utilized to refine this structure via *F*^2^-based full-matrix least squares method [18]. The compound **1ʹ**s data of crystallography and the optimization were calculated in Table 1. The chosen bond angles and bond lengths of the complex **1** are revealed in Table S1.

**Table 1.** The compound **1ʹ**s data of crystallography and the optimization of structure

Formula	C_60_H_42_Cu_3_N_4_O_16_
Fw	1265.63
Crystal system	Triclinic
Space group	*P*-1
*a* (Å)	10.6212(3)
*b* (Å)	10.9208(2)
*c* (Å)	11.7725(7)
*α*°	80.801(12)
*β*°	85.525(11)
*γ*°	70.440(10)
Volume (Å^3^)	1269.73(12)
*Z*	1
Density (calculated)	1.655
Abs. coeff. (mm^−1^)	1.327
Total reflections	9983
Unique reflections	5741
Goodness of fit on *F^2^*	1.052
Final *R* indices [*I* > 2sigma(*I*^2^)]	*R* = 0.0538, *wR*_2_ = 0.1037
*R* (all data)	*R* = 0.0879, *wR*_2_ = 0.1175
CCDC	2075258

#### Catalytic experiments

The degradation reaction was performed in a round-bottomed flask equipped with the magnetic stirrer 250 mL. In a typical Fenton reaction, 20 mg sample of **1** was dispersed into 150 mL Congo red solution with a concentration of 200 mg/L, and then it was stirred for half an hour at environmental temperature. Subsequently, 30% of (w/w) H_2_O_2_ (0.5 mL) was added into the suspension to initiate the reaction. Within a specific time interval, the residual catalyst was removed by getting out and then centrifuging the solution (3.0 mL), and in the end, it was analyzed via the ultraviolet/visible spectrophotometer. At 496 nm, the feature absorption peak was selected to monitor the degradation process. The contrast experiment in the absence of 1 was repeated at the same conditions. For the Congo red, the efficiency of degradation was calculated as follows: Degradation efficiency = (C_0_ – C_t_)/C_0_ × 100% (1), where C_t_ (mg/L) represents the concentration of Congo red at time t (min), while C_0_ (mg/L) is the initial Congo red concentration.

#### ELISA assay

The ELISA assay was conducted in this research to measure the content of TNF-α and IL-1β released into the plasma after compound treatment. This preformation was conducted totally under the guidance of the protocols with some modifications. In brief, 50 BALB/c mice (4–5 weeks, 20–22 g) were used in this research, which were divided into the model group, control group and compound treatment groups (n = 10). The stroke animal model was induced, and the new compound was given for treatment at the concentration of 1, 2 and 5 mg/kg. Then, the plasma of the animal was collected, and the content of the TNF-α and IL-1β released into the plasma was determined with three repeats.

#### Real-time RT-PCR assay

The activation of the HMGB1/TLR4 signaling pathway activation in cerebral vascular endothelial cells was further measured with real-time RT-PCR in this research, which was carried out strictly in accordance with the instructions, with only a little change. In short, 50 BALB/c mice (4–5 weeks, 20–22 g) were used in this research, which were divided into the model group, control group and compound treatment groups (n = 10). The stroke animal model was induced, and the new compound was given for treatment at the concentratiosn of 1, 2 and 5 mg/kg. Next, the cerebral vascular endothelial cells were isolated, and the total RNA in the cells was extracted. The RNA was reversed transcripted into cDNA after measuring the concentration. The relative expression of the HMGB1/TLR4 signaling pathway activation in cerebral vascular endothelial cells was measured with real time RT-PCR assay. This research was repeated at least three times, and the results were presented as mean ± SD.

## Results and discussion

### Crystal structure of compound 1

**Figure 1.**
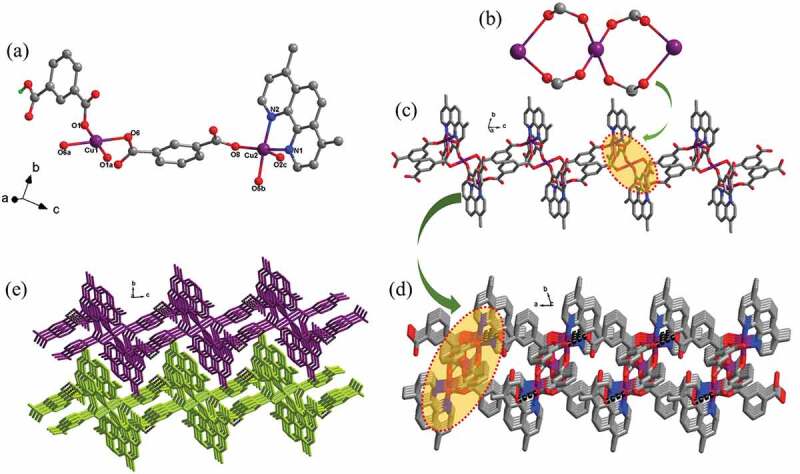
(a) The coordination surrounding view of Cu(II) ions in the complex **1.** (b) The trinuclear cluster of [Cu_3_(COO)_4_] (red: O, gray: C, purple: Cu). (c) The **1ʹ**s one-dimensional infinite chain architecture. (d) The H-bonds bridged two-dimensional layer in the molecule (red: O, gray: C, purple: Cu, blue: N). (e) Interdigitated three-dimensional supramolecular skeleton directed through the weak interactions of Van der Waals.

According to the literature results, the hydrothermal synthesis method is a feasible approach for the production of coordination polymers with high crystallinity and purity which are suitable for the single crystal X-ray studies [19–22]. The structural analysis of X-ray indicated that the complex **1ʹ**s structure reveals a one-dimensional infinite chain motif that has crystallized space groups *P*-1 of triclinic system. In its asymmetric unit, there exist 1.5 Cu(II) ions, a ligand of mbdc^2-^, a ligand of Hmbdc^−^, and one chelated dmphen ligand. The diagram of coordination surrounding of central Cu(II) ions is illustrated in the Figure 1(a). Cu1 displays an elongated octahedral coordination geometry because of the well-known effect of John-Teller; the Cu(II) reveals an asymmetric electronic configuration of d^9^. The basal plane of the octahedron is defined through four carboxylic acid O atoms coming from two ligands of mbdc^2-^ and two ligands of Hmbdc^−^, and axial sites are taken over via two carboxylic acid O atoms in two ligands of Hmbdc^−^. In basal plane, the bond distance of Cu1-O is between 1.9317(2) Å and 2.0093(2) Å, and the Cu1-O bond length in the axial direction is 2.8464(4) Å. Cu2 is five coordinated tetragonal pyramidal geometry. The basal plane of the quadrangular pyramid is defined via two carboxylic acid O atoms originating from two ligands of mbdc^2-^ and two N atoms in a chelated ligand of dmphen, and the vertex site is occupied by a carboxylic acid O atom in a ligand of Hmbdc^−^. The length of Cu2-O/N is in the range of 1.927(2)–2.295(3) Å. It is worth noting that the Cu1 ion is located in inverse center; therefore, a Cu1 ion and two symmetrically related Cu2 ions are connected through four bis-monodentate carboxylic acid groups from two mbdc^2-^ ligands and two Hmbdc^−^ ligands, giving rise to a trinuclear [Cu_3_(COO)_4_] cluster with Cu … Cu distance of 3.792 Å (Figure 1(b)). These trinuclear [Cu_3_(COO)_4_] clusters are further bridged by the mbdc^2-^ ligand into an infinite 1D chain extending along *c* direction (Figure 1(c)). In the 1D chain, the chelated dmphen ligands and Hmbdc^−^ ligands pointed outside regularly. The H donors of the undeprotonated carboxylate groups from the Hmbdc^−^ ligands can be suitably accepted via the deprotonated mbdc^2-^ carboxylic acid groups from the adjacent chain, forming nonnegligible interactions of H-bonding (with O-H … O of 2.590 Å and ∠O-H … O of 160.9°), which in depth linked these one-dimensional infinite chains into the two-dimensional layer (Figure 1(d)). In the end, these two-dimensional layers stacked together *via* the Van der Waals forces, affording an interdigitated 3D supramolecular framework (Figure 1(e)).

#### Powder X-ray diffraction (PXRD) pattern and Thermogravimetric analysis (TGA)

**Figure 2.**
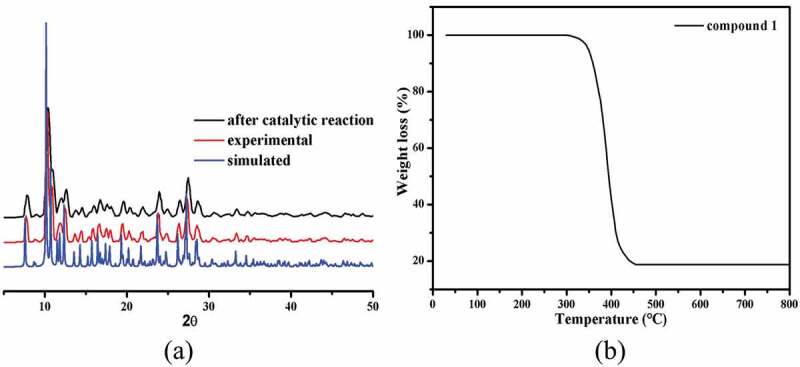
(a) The complex **1ʹ**s PXRD patterns. (b) The curve of TGA for the complex **1**.

The generated massive samples’ phase purity was proved through the study of PXRD, and the PXRD manners of simulation and experiment are reflected in Figure 2(a). Significantly, the diffraction peaks for the patterns of experiment are in accordance with that of the patterns of simulation, revealing the polycrystalline samples are of single phase.

In addition, the experiment of TGA was also implemented to explore the **1ʹ**s thermal stability. The complex **1ʹ**s curve of TGA shows only an obvious and rapid process of weightlessness between 297 °C and 455 °C, which on account of the organic ligands decomposition and the final residues with a weight of 18.73% may be assigned to the generation of the CuO (with 18.96% calculated value) (Figure 2(b)). Considering the following bioactivity tests, it is necessary to study the structural stability of **1** in the dispersing solvents DMSO. It is well known that coordination polymers could not be dissolved in common solvents, so we used their stock solution in the following bioactivity tests which has been well documented in the literatures [23,24]. First, a small amount (nearly 150–160 mg) of the synthesized coordination polymer was taken in a mortar. It was then ground manually for 30 min by using a pestle to obtain the fine powders. After that, the fine powders were soaked in 50 mL DMSO and subjected to the ultrasonic treatments for 2 hours at the powder of 70 W to get the well-dispersed stock solution. The FT-IR data show that the structure of complex **1** did not change in the stock solutions, indicating its considerable framework stability (Fig S1).

#### Catalytic properties of 1

**Figure 3.**
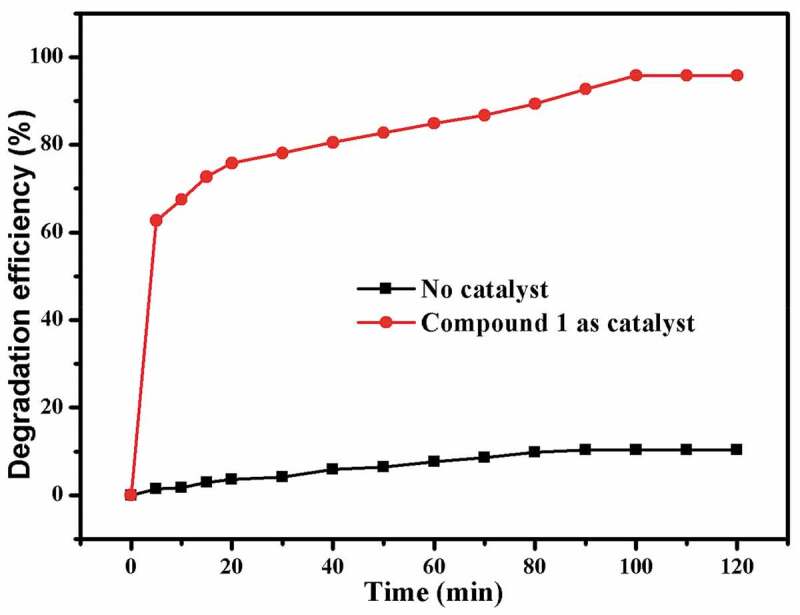
The outcomes of experiment of the Congo red catalytic degradation.

As we all know, organic azo dyes are widely used in the fields of pharmaceutical, textile, food and cosmetics industries [25]. The discharge of wastewater containing organic azo dyes will cause serious hazard to human health and ecological environment owing to their complex aromatic structures that are highly resistant to conventional wastewater treatment process [26]. To reduce the damage of organic dyes to environment, it is a requirement for us to develop an alternative technique that can effectively degrade the organic dyes in waste water. Recently, oxidization technology on the basis of the reaction of Fenton has attracted great interest owing to their huge potential to completely mineralize organic azo dyes. In accordance with the previously reported literatures, it can be observed that •OH radicals are the main active species produced by H_2_O_2_ with the aid of the catalyst in a reaction of Fenton. In our investigation, the Congo red was chosen as the representative to evaluate the catalytic activity of **1**, and the Congo red degradation research was implemented with the aid of H_2_O_2_ through a Fenton reaction. The degradation efficiencies of Congo red with or without catalysts are shown in Figure 3. When using compound **1** as catalyst, the degradation efficiency rapidly reached 62.7% within 5 min, and then the degradation reaction rate slowed down. At 100 min, the degradation efficiency reached the maximum of 95.8%, and after that, the degradation reaction almost stopped with no appreciable increase of degradation efficiency. The concentration of the Cu^2+^ ion in the solution was studied by the ICP-AES (inductively coupled plasma–Atomic emission spectrometry analysis), which gave a value of 6.28 mg/L, indicating little Cu^2+^ ion was leached from the framework to the solution. The contrast experiment in absence of **1** was also performed at various conditions, and the efficiency of degradation is only 10.32% within 120 min. Obviously, these results demonstrated that compound **1** displays high catalytic effect for the Congo red degradation in a reaction of Fenton. The possible reaction mechanism for this reaction is similar to that of a previously reported literature [27]. After degradation reaction, the structure of **1** remains unchanged compared to the pristine sample that was confirmed by the PXRD experimental (Figure 2(a)).

#### Compound obviously reduced the releasing of TNF-α and IL-1β into the plasma

**Figure 4.**
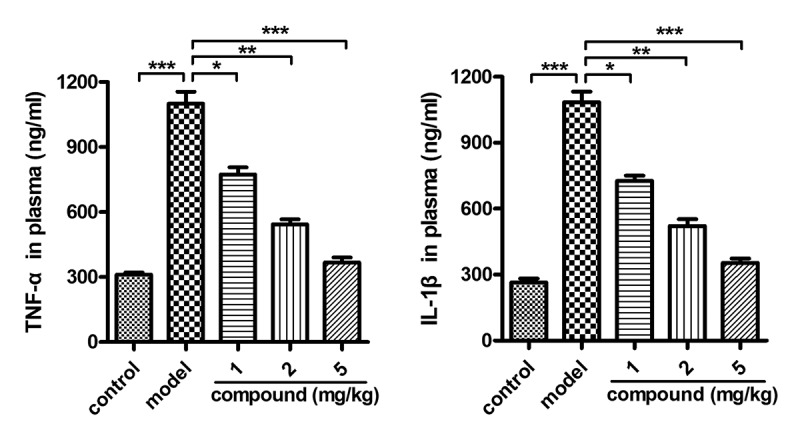
Obviously reduced releasing of TNF-α and IL-1β into the plasma after compound treatment. The stroke animal model was constructed, and the compound was given for the indicated treatment. The TNF-α and IL-1β content released into the plasma was determined with ELISA assay.

After the synthesis of the new compound with novel structure, its application value on stroke was evaluated, and the related mechanism was explored at the same time. Thus, the ELISA assay was first conducted and the content of TNF-α and IL-1β released into the plasma was determined. The results in Figure 4 suggested that there was higher level of the TNF-α and IL-1β in the model group compared with the control group. There was a significant difference between these two groups, with P < 0.005. After the treatment of the new compound, the level of the TNF-α and IL-1β released into the plasma was significantly reduced. The inhibition of the new compound exhibited a dose-dependent manner.

#### Compound significantly inhibited the activation of the HMGB1/TLR4 signaling pathway activation in cerebral vascular endothelial cells

**Figure 5.**
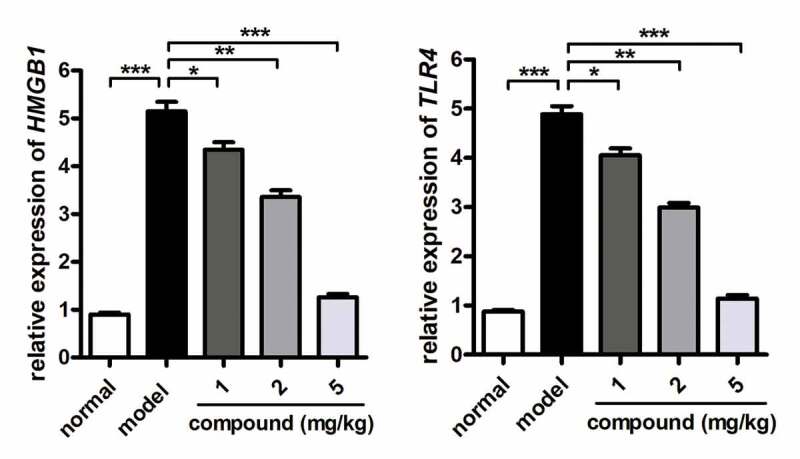
Significantly inhibited activation of the HMGB1/TLR4 signaling pathway activation in cerebral vascular endothelial cells after compound treatment. The stroke animal model was constructed, and the compound was given for indicated treatment. The activation of the HMGB1/TLR4 signaling pathway activation in cerebral vascular endothelial cells was determined with RT-PCR assay.

In the above research, we have proved that the new compound could significantly reduce the releasing of TNF-α and IL-1β into the plasma in a dose-dependent manner. The HMGB1/TLR4 signaling pathway was also important for the development of stroke disease. So, the real-time RT-PCR was further conducted, and the results shown in Figure 5 suggested that the activation of HMGB1/TLR4 signaling pathway activation in the model group was much higher than that of the control group. Under the treatment of the compound, the HMGB1/TLR4 signaling pathway activation was obviously reduced in a dose-dependent manner. This result was consistent with the above research in Figure 4.

## Conclusions

In summary, employing the dual-ligand strategy, we obtained a fresh Cu(II) coordination polymer, and it possesses a 1D chain structure with trinuclear [Cu_3_(COO)_4_] clusters. Intermolecular hydrogen bonds further bridged these one-dimensional chains into the two-dimensional layer, and in the end, these two-dimensional layers are in-depth stacked into the interdigitated three-dimensional supramolecular skeleton *through* the weak interactions of Van der Waals. This compound possesses high thermal stability and exhibits high catalytic effect for the Congo red degradation in a reaction of Fenton. The application value of the compound on stroke was assessed, and the results of ELISA assay suggested that the compound could obviously reduce the releasing of the TNF-α and IL-1β into the plasma in a dose-dependent manner. In addition to this, the activation of the HMGB1/TLR4 signaling pathway activation in cerebral vascular endothelial cells was also obviously reduced by the new compound dose dependently. In the end, we draw this conclusion, the new compound has excellent application value on the stroke therapy by inhibiting the inflammatory response in the cerebral vascular endothelial cells.

## Data Availability

The table reflecting the bond angles and bond lengths for the complex **1** (Table S1); the FT-IR spectra of complex **1** (Fig. S1), and the information could be found in the supporting information file.
